# Usefulness of ultrasonography and elastography in diagnosing oxaliplatin-induced sinusoidal obstruction syndrome

**DOI:** 10.1007/s10147-022-02235-4

**Published:** 2022-08-30

**Authors:** Rika Saito, Yasuyuki Kawamoto, Mutsumi Nishida, Takahito Iwai, Yasuka Kikuchi, Isao Yokota, Ryo Takagi, Takahiro Yamamura, Ken Ito, Kazuaki Harada, Satoshi Yuki, Yoshito Komatsu, Naoya Sakamoto

**Affiliations:** 1 Division of Cancer Center, Hokkaido University Hospital, Kita-15, Nishi-7, Kita-ku, Sapporo, Japan; 2 Diagnostic Center for Sonography, Hokkaido University Hospital, Sapporo, Japan; 3 Department of Diagnostic Imaging, Faculty of Medicine, Hokkaido University, Sapporo, Japan; 4 Department of Biostatistics, Graduate School of Medicine, Hokkaido University, Sapporo, Japan; 5 Department of Gastroenterology and Hepatology, Hokkaido University Hospital, Sapporo, Japan

**Keywords:** Ultrasonography, Liver stiffness measurement, Shear-wave elastography, Oxaliplatin, Sinusoidal obstruction syndrome

## Abstract

**Background:**

Sinusoidal obstruction syndrome (SOS) refers to liver injury caused by hematopoietic stem cell transplantation (HSCT) and anticancer drugs including oxaliplatin. Increased splenic volume (SV) on computed tomography (CT) indicates oxaliplatin-induced SOS. Similarly, ultrasonography and liver stiffness measurement (LSM) by shear-wave elastography (SWE) can help diagnose SOS after HSCT; however, their usefulness for diagnosing oxaliplatin-induced SOS remains unclear. We investigated the usefulness of the Hokkaido ultrasonography-based scoring system with 10 ultrasonographic parameters (HokUS-10) and SWE in diagnosing oxaliplatin-induced SOS early.

**Methods:**

In this prospective observational study, ultrasonography and SWE were performed before and at 2, 4, and 6 months after oxaliplatin-based chemotherapy. HokUS-10 was used for assessment. CT volumetry of the SV was performed in clinical practice, and an SV increase ≥ 30% was considered the diagnostic indicator of oxaliplatin-induced SOS. We assessed whether HokUS-10 and SWE can lead to an early detection of oxaliplatin-induced SOS before an increased SV on CT.

**Results:**

Of the 30 enrolled patients with gastrointestinal cancers, 12 (40.0%) with an SV increase  ≥ 30% on CT were diagnosed with SOS. The HokUS-10 score was not correlated with an SV increase ≥ 30% (*r* = 0.18). The change in rate of three HokUS-10 parameters were correlated with an SV increase ≥ 30% (*r* = 0.32–0.41). The change in rate of LSM by SWE was correlated with an SV increase  ≥ 30% (*r* = 0.40).

**Conclusions:**

The usefulness of HokUS-10 score was not demonstrated; however, some HokUS-10 parameters and SWE could be useful for the early diagnosis of oxaliplatin-induced SOS.

**Supplementary Information:**

The online version contains supplementary material available at 10.1007/s10147-022-02235-4.

## Introduction

Hepatic sinusoidal obstruction syndrome (SOS), also known as central hepatic veno-occlusive disease, is caused by non-thrombotic obstruction of the central and sublobular hepatic veins that congests the hepatic sinusoids and leads to necrosis of the surrounding hepatocellular tissue. SOS ultimately results in ascites, painful hepatomegaly, jaundice, and elevated serum aspartate aminotransferase (AST) and alanine aminotransferase (ALT) levels [1–3]. Hematopoietic stem cell transplantation (HSCT), radiotherapy, and anticancer drugs trigger SOS [4, 5]. SOS, induced by the anticancer drug oxaliplatin, a third-generation platinum drug that is widely used in gastrointestinal cancers, was first reported in 2004 [6, 7]. The incidence of oxaliplatin-induced SOS varies from 19 to 78% [6, 8–14]. In SOS, hepatic sinusoid obstruction causes portal hypertension, leading to splenomegaly and thrombocytopenia [13]. Oxaliplatin-induced SOS increases postoperative mortality, blood transfusion requirements, and hospital stay after the resection of liver metastasis in colorectal cancer [8, 10, 11]; it also increases early postoperative recurrence and decreases long-term survival rates [15]. Furthermore, it decreases chemotherapeutic effects [16] and complicates chemotherapy continuation of due to thrombocytopenia. Thus, a method that can diagnose oxaliplatin-induced SOS, early, is required.

Currently, increased splenic volume (SV) on computed tomography (CT) has become a common indicator of oxaliplatin-induced SOS [13, 17–20]. An increase in SV correlates with histopathological liver sinusoidal endothelial cell damage, and an SV increase ≥ 30% on CT is an independent predictor for oxaliplatin-induced SOS, with specificity of 71–100% [13, 17, 21]. Recently, oxaliplatin-induced SOS risk was evaluated using increased SV as a diagnostic indicator [22, 23]. However, the median time to an increase SV ≥ 30% on CT was 5.4 (95% confidence interval [CI], 4.8–6.5) months [23]; thus requiring at least 6 months for diagnosis. Although increased SV on CT is a useful diagnostic indicator, an earlier diagnostic imaging method is required to identify oxaliplatin-induced SOS. Histological diagnosis is a robust method for detecting oxaliplatin-induced SOS; however, liver biopsies are difficult in patients, because they can be over-invasive, depending on the patient's general condition.

To develop a novel early diagnostic method for oxaliplatin-induced SOS, we focused on abdominal ultrasonography and elastography that were useful for diagnosing SOS after HSCT. Compared to CT, ultrasonography and elastography are advantageous in terms of cost and exposure risk. Ultrasonography with Doppler imaging is also beneficial for detecting blood flow abnormalities in SOS [24–30]. Using previous reports, we extracted 10 diagnostically useful ultrasonography parameters, named the Hokkaido ultrasonography-based scoring system (HoKUS-10) [31–33]. The HokUS-10 with 100% sensitivity and 95.8% specificity [33], is expected to be widely used as part of the diagnostic imaging for SOS after HSCT. SOS occurring after HSCT generally develops within 21 days of HSCT (also called classical SOS) [34]. However, oxaliplatin-induced SOS may develop over a more chronic course than the classical SOS. Presently, no reports have investigated whether HokUS-10 is useful for diagnosing oxaliplatin-induced SOS early.

Elastography, a method used to measure tissue elasticity, can directly assess liver fibrosis and is a useful noninvasive alternative to liver biopsy. It works based on the principle of an excitation method (acoustic radiation force impulse or mechanical impulse) and the measured physical quantity (strain or shear-wave transmission velocity) [35]. Shear waves are transverse waves that travel inside an object, generated by applying constant ultrasonic wave compression. An object’s stiffness can be measured by calculating its shear-wave transmission velocity. Elastographies that use the principles of measuring shear-wave transfer velocities include shear-wave elastography (SWE) and transient ultrasonography. SWE generates shear waves by continuously irradiating ultrasonic waves, and the measured velocity can be superimposed with the image to display color mapping; hence, the stiffness can be visually inferred. The usefulness of SWE in diagnosing SOS after HSCT has been reported [36, 37].

In this study, we aimed to investigate the usefulness of HokUS-10 and SWE in the early diagnosis of oxaliplatin-induced SOS. We investigated whether preplanned ultrasonography and SWE assessments could be used to screen for SOS before CT can detect an increase in SV.

## Patients and methods

### Patients

This prospective observational study was conducted between December 2019 and June 2021 at Hokkaido University Hospital. The inclusion criteria were as follows: patients with unresectable advanced or recurrent colorectal, pancreatic, gastric, and esophageal cancers scheduled to receive oxaliplatin-based chemotherapy; resected colorectal cancer scheduled to receive adjuvant oxaliplatin-based chemotherapy; aged ≥ 20 years, and expected to survive for at least 6 months from the registration date. The exclusion criteria were as follows: patients with liver metastasis; history of hepatectomy or splenectomy; comorbidity with disorders that increase portal pressure (including cirrhosis, chronic hepatitis, right heart failure, and tumor vascular involvement); and history of oxaliplatin-based chemotherapy. All included patients provided written informed consent in accordance with the Declaration of Helsinki. The institutional review board of Hokkaido University Hospital approved this study (approval number: 019-0133).

### Study design

Ultrasonography and SWE were performed before and at 2, 4, and 6 months after the initiation day of oxaliplatin-based chemotherapy (the acceptable time for examination was ± 4 weeks). CT was performed on the same day as ultrasonography and SWE as much as possible in clinical practice, including 6 months after treatment initiation. In this study, an SV increase ≥ 30% on CT after oxaliplatin-based chemotherapy was defined as the diagnostic indicator of SOS [13, 17, 21, 23]. The following patient data were collected: age, sex, Eastern Cooperative Oncology Group (ECOG) performance status, body mass index (BMI), primary site, regimen, duration of oxaliplatin use, cumulative oxaliplatin dose, relative oxaliplatin dose intensity (RDI), and laboratory values (platelet count, serum total bilirubin levels, AST, ALT, serum alkaline phosphatase [ALP], serum γ-glutamyltransferase [γ-GT]), and serological marker, such as AST to platelet ratio index (APRI) [38, 39].

### Assessment of ultrasonography and elastography

The HokUS-10 score was used to assess ultrasonography for a diagnosing cutoff value for SOS ≥ 5 points [33] (Table 1, Figure S1). We used an Aplio-i700/800 device (Canon Medical Systems Corp., Otawara, Japan) and convex (4.75 and 6.0 MHz) and linear probes (7.5 MHz) for the B-mode and color doppler assessments.

**Table 1:** HokUS-10 scoring system

	Parameter	Description	Score
1	Hepatic left lobe vertical diameter (a)	≥ 70 mm	1
2	Hepatic right lobe vertical diameter (b)	≥ 110 mm	1
3	Gallbladder wall thickening (c)	≥ 6 mm	1
4	PV diameter (d)	≥ 12 mm	1
5	PUV diameter (e)	≥ 2 mm	2
6	Amount of ascites (f)	Mild	1
		Moderate to severe	2
7	PV mean velocity (g)	< 10 cm/s	1
8	Direction of the PV blood flow signal (h)	Congestion or hepatofugal	1
9	Appearance of the PUV blood flow signal (i)	Yes	2
10	Hepatic artery resistive index (j)	≥ 0.75	1
Total			13

*PV* portal vein, *PUV* paraumbilical vein

2D-SWE, used for assessing elastography, was performed at the same time as the ultrasonography. The field of view was set at 1–2 cm below the hepatic capsule, and a 10 mm-diameter spherical region of interest was located, then the LSM was performed. Measurements were conducted at least five times in the right intercostal scan at sites without vessels or masses in the right liver lobe, and the median measurement value (m/s) was calculated. Values were considered reliable if the ratio of the interquartile range to the median was ≤ 30%.

Patients fasted for > 4 h prior to ultrasonography and elastography. Both procedures were performed by a gastroenterologist with 8 years of experience and two registered medical sonographers with 10 and 36 years of experience, respectively. All measurements by the gastroenterologist were double-checked by a registered senior medical sonographer. Regarding elastography reproducibility, the examination order was not fixed, and the measurement was conducted independently.

### Assessment of SV on CT

CT was performed with > 160 slices, and reconstruction was performed in 5-mm slices. SV was measured using the volume calculator SYNAPSE VINCENT v5.3^®^ (Fujifilm, Tokyo, Japan). Imaging was performed by a radiologist.

### Statistical analysis

Considering the feasibility of this study, we set the sample size to 30. The statistical power was 0.8, the proportion of patients with an SV increase ≥ 30% was 0.75 and 0.25 in those with HokUS-10 scores ≥ 5 and < 5, respectively, assuming a proportion of patients with HokUS-10 ≥ 5 of 0.5 in the population. Based on the above, we assumed a risk ratio ≥ 3.0 to be useful for diagnosing oxaliplatin-induced SOS. In this study, when SV increase ≥ 30% was observed, the score of HoKUS-10 just before SV increase ≥ 30% was used for analysis. When SV increase ≥ 30% was not observed, the score of HokUS-10 just before the largest SV increase during observation period was used in the analysis. LSM, laboratory values and the values of serological marker were also analyzed by the same method to assess the ability to predict oxaliplatin-induced SOS. The recruitment period was from December 2019 to December 2020.

The RDI of oxaliplatin was defined as the actual administered divided by the scheduled dose intensity. The dose intensity was the total dose divided by the number of days of oxaliplatin use. The mean and difference between those with and without an SV increase ≥ 30% was estimated with a 95% CI based on Wilcoxon’s rank sum test. The proportion with an SV increase ≥ 30% was classified as HokUS-10 ≥ 5 or < 5 according to the risk ratio and 95% CI. We also calculated the Spearman’s rank correlation coefficient between SV increase and HokUS-10 score. We defined the change in rate as ([post-measurement–pre-measurement]/pre-measurement × 100) before and after oxaliplatin-based chemotherapy. We evaluated the Pearson’s correlation coefficient between the SV increase and the change in rate of seven continuous parameters (left and right lobe vertical diameter, gallbladder wall thickening, portal vein (PV) diameter, paraumbilical vein (PUV) diameter, PV mean velocity, and hepatic artery resistive index [(RI)] of HokUS-10. We estimated the Pearson’s correlation coefficient between SV increase and change in rate of LSM evaluated by SWE. Regarding inter-examiner reproducibility of LSM, we measured the intraclass correlation coefficients (ICC) and conducted a Bland–Altman analysis. The association of SV increase ≥ 30% with laboratory values and APRI (AST [IU/L]/ upper limit of normal of AST[IU/L]/platelet count [10^9^/L] × 100), was also investigated [38, 39].

All statistical data were analyzed by a two-sided test and *p* values < 0.05 was considered significant, using JMP^®^ 14 (SAS Institute Inc., Cary, NC, USA).

## Results

### Patient characteristics and profiles

**Fig. 1:**
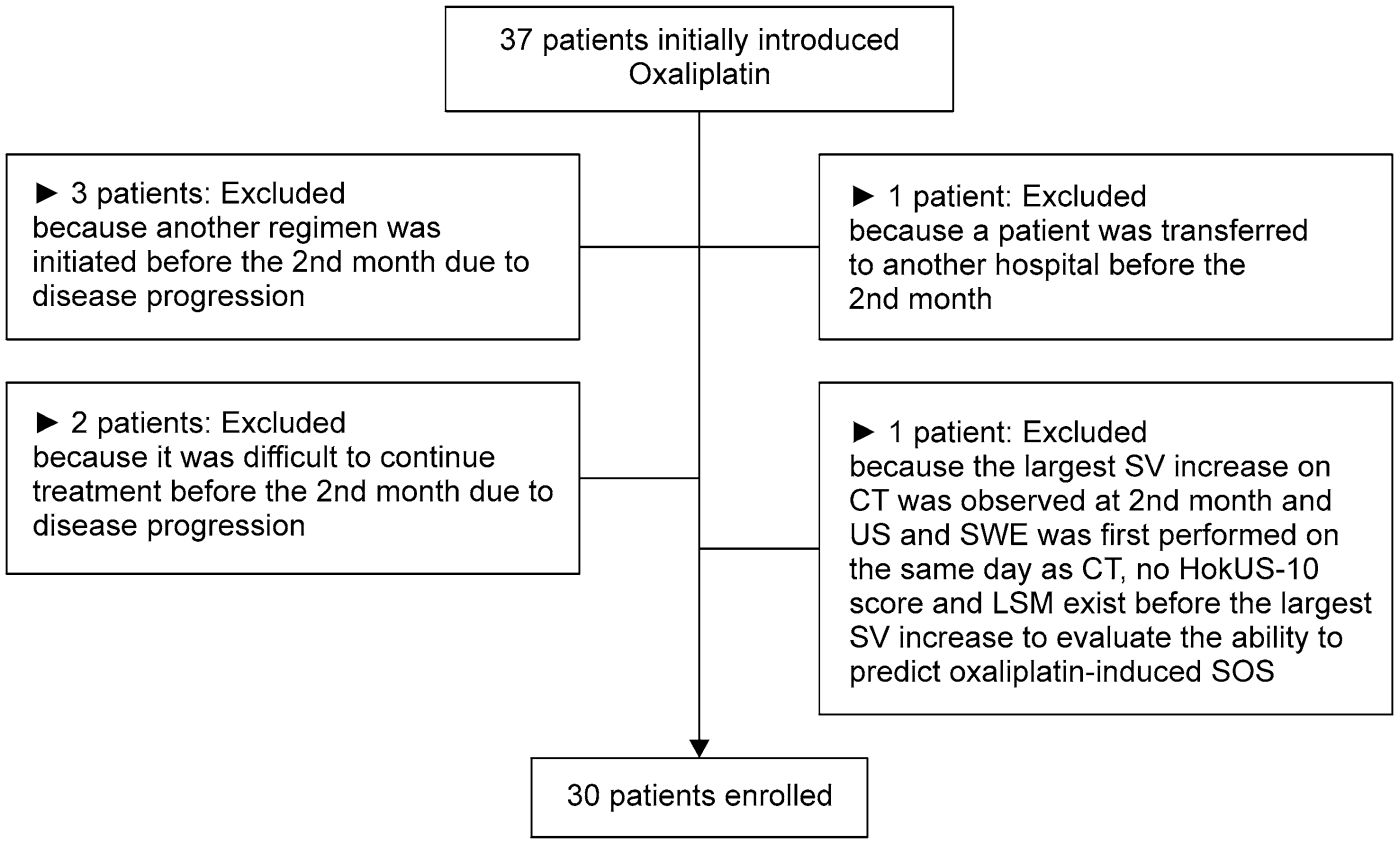
Flowchart of patient selection 37 patients initially were enrolled. One patient was excluded, because the largest splenic volume (SV) increase on computed tomography (CT) was observed at 2nd month and ultrasonography (US) and shear-wave elastography (SWE) was first performed on the same day as CT, no score of Hokkaido ultrasonography-based scoring system with 10 ultrasonographic parameters (HokUS-10) and liver stiffness measurement (LSM) exist before the largest SV increase to evaluate the ability to predict oxaliplatin-induced sinusoidal obstruction syndrome (SOS). A total of seven patients including the above one patient did not meet the inclusion criteria and were, therefore, excluded from the analysis.

A total of 37 patients were enrolled, but seven did not meet the inclusion criteria and were excluded. Finally, 30 patients were evaluated (Fig. 1).

Table 2 shows the characteristics of the patients, including 14 male and 16 female with a median age of 63 (range, 42–83) years. The ECOG PS was 0, 1, and 2 in 25 (84%), 4 (13%), and 1 (3%) patient, respectively. The median BMI was 22.0 ± 4.2 kg/m^2^. The most common cancer was colorectal cancer (16 [53%]), followed by pancreatic (6 [20%]), gastric (6 [20%]), and esophageal (2 [7%]) cancers. Table 2 summarizes the chemotherapy regimens, duration and dose of oxaliplatin, the measurement of each HokUS-10 parameter, liver stiffness measurement and laboratory data before oxaliplatin-based chemotherapy.

**Table 2:** Patient characteristics

	*N* (%)
Age (years, range)	63 (42–83)
Sex	
Male	14 (47%)
Female	16 (53%)
ECOG^a^ performance status	
0	25 (83%)
1	4 (13%)
2	1 (4%)
BMI (kg/m^2^, mean ± SD)	22.0±4.2
Primary site	
Colorectal cancer	16 (53%)
Pancreatic cancer	6 (20%)
Gastric cancer	6 (20%)
Esophageal cancer	2 (7%)
Regimen	
CapeOX	16 (53%)
mFOLFOX6	5 (17%)
FOLFIRINOX	3 (10%)
OX-IRIS	3 (10%)
SOX	2 (7%)
CapeOX + Tmab	1 (3%)
Oxaliplatin use	
Duration of use (days)	97 (21–213)
Cumulative dose (mg/m^2^, mean ± SD)	603.6 ± 246.5
Relative dose intensity (%, mean ± SD)	85.1 ± 15.0
Measurement of each HokUS-10 parameter before oxaliplatin-based chemotherapy (mean ± SD)	
Left lobe vertical diameter (mm)	53.2 ± 13.3
Right lobe vertical diameter (mm)	106.4 ± 16.7
Gallbladder wall thickening (mm)	1.17 ± 0.29
Portal vein diameter (mm)	10.3 ± 2.30
Paraumbilical vein diameter (mm)	1.09 ± 0.36
Portal vein mean velocity (m/s)	16.6 ± 4.37
Hepatic artery resistive index	0.72 ± 0.07
Liver stiffness measurement (mean ± SD)	
Liver stiffness measurement (m/s)	1.27 ± 0.12
Laboratory values (mean ± SD)	
Platelet count (× 10^9^/L)	273.7 ± 73.7
Total bilirubin level (mg/dL)	0.64 ± 0.17
AST (IU/L)	21.7 ± 8.9
ALT^k^ (IU/L)	18.8 ± 9.6
ALP^l^ (IU/L)	286.3 ± 147.8
γ-GT^m^ (IU/L)	44.5 ± 50.5

*ECOG* Eastern cooperative oncology Group*BMI *body mass index, *SD* standard deviation, *CapeOX* capecitabine and oxaliplatin therapy, *mFOLFOX6* oxaliplatin, levofolinate, and 5-FU therapy, *FOLFIRINOX* oxaliplatin, irinotecan, levofolinate, and 5-FU therapy, OX-IRIS, oxaliplatin, irinotecan, and TS-1 therapy, *SOX* TS-1 and oxaliplatin therapy, *Tmab* trastuzumab, *AST* serum aspartate aminotransferase, *ALT* serum alanine aminotransferase, ALP serum alkaline phosphatase, *γ-GT*, serum γ-glutamyltransferase

**Fig. 2:**
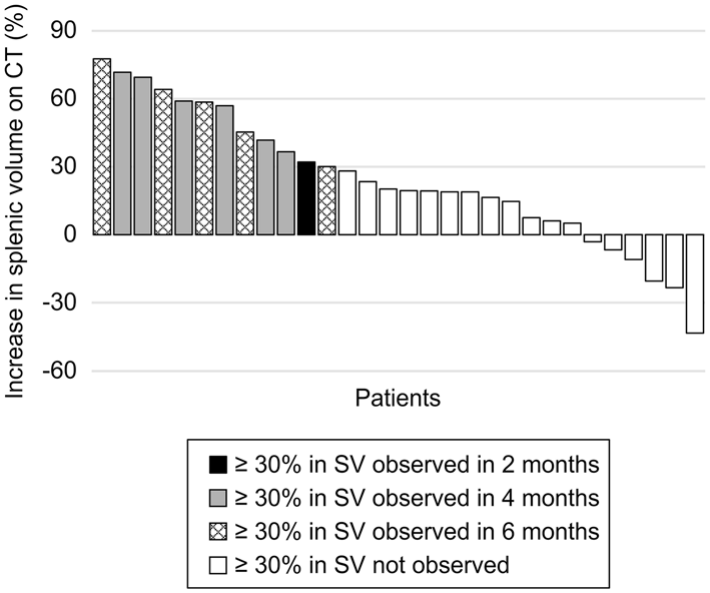
Increase in splenic volume on CT The change in splenic volume (SV) compared to SV before oxaliplatin-based chemotherapy is shown for each patient. Patients are color-coded according to the time point at which an increase ≥ 30% in SV on computed tomography (CT) was observed (2 months, black; 4 months, gray; 6 months, shaded). Patients without an increase ≥ 30% in SV are coded as white.

Of the 30 patients, 12 (40.0%) had an SV increase ≥ 30% after commencing oxaliplatin-based chemotherapy (Fig. 2). We found no differences in age, sex, ECOG PS, BMI, primary site, regimens when comparing 12 patients with and 18 without an SV increase ≥ 30% (Table 3). The duration of oxaliplatin use and mean RDI of oxaliplatin were not significantly different; however, the cumulative dose of oxaliplatin was significantly higher in patients with an SV increase of ≥ 30% (*p* = 0.03). There were no significant differences in measurement of each HokUS-10 parameter, LSM and laboratory values before oxaliplatin-based chemotherapy between patients with and without an SV increase ≥ 30%.

**Table 3:** Patient profile

	≥ 30% increase in splenic volume		*p* value
	Observed	Not observed	
	(*N* = 12)	(*N* = 18)	
Age (years, range)	62 (45–72)	64 (42–83)	0.41^*^
Sex			
Male	5 (42%)	9 (50%)	
Female	7 (58%)	9 (50%)	0.72^†^
ECOG performance status			
0	12 (100%)	13 (72%)	
1	0 (0%)	4 (22%)	
2	0 (0%)	1 (6%)	0.18^†^
BMI (kg/m^2^, mean ± SD)	22.9±4.8	21.5 ± 3.8	0.51^*^
Primary site			
Colorectal cancer	7 (59%)	9 (50%)	
Pancreatic cancer	1 (8%)	5 (28%)	
Gastric cancer	3 (25%)	3 (17%)	
Esophageal cancer	1 (8%)	1 (5%)	0.64^†^
Regimen			
CapeOX	7 (58%)	9 (50%)	
mFOLFOX6	2 (18%)	3 (17%)	
FOLFIRINOX	0 (0%)	3 (17%)	
OX-IRIS	1 (8%)	2 (11%)	
SOX	1 (8%)	1 (5%)	
CapeOX + Tmab	1 (8%)	0 (0%)	0.64^†^
Oxaliplatin use			
Duration of use (days)	139 (63–175)	71(21–231)	0.09^*^
Cumulative dose (mg/m^2^, mean ± SD)	719.8 ± 66.6	526.1 ± 54.4	0.03^*^
Relative dose intensity (%, mean ± SD)	83.4 ± 4.4	86.2 ± 3.6	0.47^*^
Measurement of each HokUS-10 parameter before oxaliplatin-based chemotherapy (mean ± SD)			
Left lobe vertical diameter (mm)	56.4 ± 15.3	51.2 ± 12.0	0.51^*^
Right lobe vertical diameter (mm)	104.2 ± 15.7	107.9 ± 17.7	0.57^*^
Gallbladder wall thickening (mm)	1.16 ± 0.24	1.17 ± 0.32	0.73^*^
Portal vein diameter (mm)	10.3 ± 2.50	10.4 ± 2.23	0.69^*^
Paraumbilical vein diameter (mm)	1.18 ± 0.37	1.04 ± 0.36	0.31^*^
Portal vein mean velocity (m/s)	16.1 ± 4.97	17.0 ± 4.04	0.50^*^
Hepatic artery resistive index	0.71 ± 0.07	0.72 ± 0.07	0.80^*^
Liver stiffness measurement before oxaliplatin-based chemotherapy (mean ± SD)			
Liver stiffness measurement (m/s)	1.24 ± 0.04	1.29 ± 0.03	0.30^*^
Laboratory values before oxaliplatin-based chemotherapy (mean ± SD)			
Platelet count (× 10^9^/L)	283.7 ± 21.5	267.1 ± 17.6	0.46^*^
Total bilirubin level (mg/dL)	0.68 ± 0.05	0.61 ± 0.04	0.30^*^
AST (IU/L)	21.4 ± 2.6	22.0 ± 2.1	0.34^*^
ALT (IU/L)	17.3 ± 2.8	19.8 ± 2.3	0.51^*^
ALP (IU/L)	321.2 ± 44.6	264.9 ± 34.8	0.74^*^
γ-GT (IU/L)	31.8 ± 14.4	54.7 ± 11.8	0.37^*^

^*^Wilcoxon’s rank sum test

^†^Fisher’s exact test

*ECOG* eastern cooperative oncology group, *BMI* Body mass index, *SD* standard deviation, *CapeOX* capecitabine and oxaliplatin therapy, *mFOLFOX6* oxaliplatin, levofolinate, and 5-FU therapy, *FOLFIRINOX* oxaliplatin, irinotecan, levofolinate, and 5-FU therapy, *OX-IRIS* oxaliplatin, irinotecan, and TS-1 therapy, *SOX* TS-1 and oxaliplatin therapy, *Tmab* trastuzumab, *AST* serum aspartate aminotransferase, *ALT* serum alanine aminotransferase, *ALP* serum alkaline phosphatase, *γ-GT* serum γ-glutamyltransferase

### Correlation between HokUS-10 and SV increase

The proportions of SV increase ≥ 30% occurred in 100% (1/1 patients) and 37.9% (11/29 patients) in the HokUS-10 ≥ 5 and < 5 groups, respectively, while the risk ratio was 2.64 (95% CI 1.66–4.20) (Table 4). Correlation between the HokUS-10 score and SV increase was not apparent (Spearman’s rank correlation coefficient, 0.18). We also summarized the presence of an SV increase ≥ 30% and each HokUS-10 parameter score (Table S1, Supplementary Data). The risk ratios for all 10 parameters were < 3.0, and no correlation was shown with an SV increase ≥ 30%.

**Table 4:** Cross-tabulation of the HokUS-10 scoring system

HokUS-10	≥ 30% increase in splenic volume		Total
	Observed	Not observed	(*n*)
Score ≥ 5	1	0	1
Score < 5	11	18	29
Total (*n*)	12	18	30

**Fig. 3:**
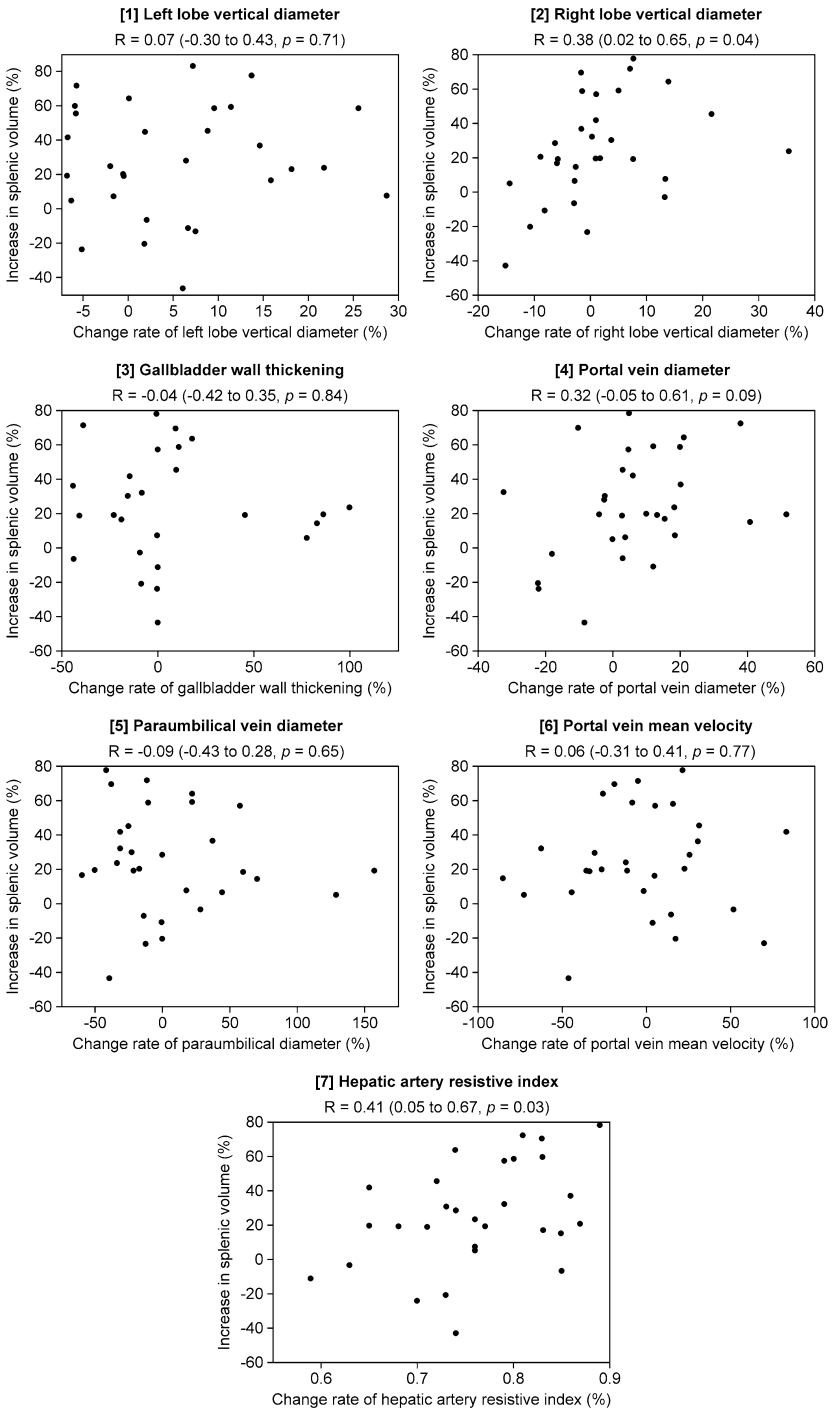
Scatter plots and correlation coefficients for the change in rate of HokUS-10 parameters and the increase in splenic volume Pearson's correlation coefficients (R 95% Confidence interval, CI) were calculated for the change in rate of the continuous seven HokUS-10 parameters ([1] left lobe vertical diameter, [2] right lobe vertical diameter, [3] gallbladder wall thickening, [4] portal vein diameter, [5] paraumbilical vein diameter, [6] portal vein mean velocity, and [7] hepatic artery resistive index) and increase in splenic volume.

Next, we evaluated the correlation between the change in rate of seven continuous HokUS-10 parameters (left and right lobe vertical diameter, gallbladder wall thickening, PV diameter, PUV diameter, PV mean velocity, and hepatic artery RI) with SV increase (Table 5). The change in rate of right lobe vertical diameter was significantly increased patients with an SV increase ≥ 30% (*p* = 0.03). Among the seven continuous parameters, weak Pearson's correlation coefficients’ (|*r|*= 0.2–0.4) was found in two parameters (right lobe vertical diameter: *r* = 0.38; PV diameter: *r* = 0.32); and moderate Pearson's correlation coefficients’ (|*r*|= 0.4–0.7) was found in one parameter (hepatic artery RI: *r* = 0.41) (Fig. 3).

**Table 5:** Change rate and values of measurements and increase ≥ 30% in splenic volume

	≥ 30% increase in splenic volume		*p* value
	Observed (*N* = 12)	Not observed (*N* = 18)	
Change rate of each HokUS-10 parameter (mean ± SD)			
Left lobe vertical diameter (%)	1.78 ± 11.0	− 0.87 ± 12.1	0.67^*^
Right lobe vertical diameter (%)	4.77 ± 7.09	− 0.57 ± 12.1	0.03^*^
Gallbladder wall thickening (%)	− 6.39 ± 20.2	16.8 ± 48.4	0.62^*^
Portal vein diameter (%)	7.15 ± 17.8	6.34 ± 19.4	0.57^*^
Paraumbilical vein diameter (%)	− 6.09 ± 32.7	14.4 ± 58.9	0.41^*^
Portal vein mean velocity (%)	3.04 ± 37.5	− 9.04 ± 40.5	0.39^*^
Hepatic artery resistive index (%)	0.79 ± 0.07	0.74 ± 0.08	0.14^*^
Change rate of liver stiffness measurement (mean ± SD)			
Liver stiffness measurement (%)	18.7 ± 3.92	8.05 ± 3.20	0.04^*^
Changes in value of serological markers (mean ± SD)			
Platelet count (× 10^9^/L)	− 180.0 ± 19.4	− 109.1 ± 15.1	0.01^*^
Total bilirubin level (mg/dL)	0.31 ± 0.11	0.18 ± 0.09	0.27^*^
AST (IU/L)	20.3 ± 6.0	13.1 ± 4.7	0.18^*^
ALT (IU/L)	20.5 ± 9.7	22.2 ± 7.9	0.42^*^
ALP (IU/L)	− 9.5 ± 50.4	17.4 ± 37.6	0.77^*^
γ-GT (IU/L)	11.2 ± 17.1	47.8 ± 13.4	0.17^*^
Serological marker (mean ± SD)			
APRI^f^	1.13 ± 0.19	0.68 ± 0.15	0.046^*^

*SD* standard deviation, *AST* serum aspartate aminotransferase, *ALT* serum alanine aminotransferase, *ALP* serum alkaline phosphatase, *γ-GT* serum γ-glutamyltransferase, *APRI* serum aspartate aminotransferase to platelet ratio index

^*^Wilcoxon’s rank sum test

### Correlation between LSM and increase in SV

**Fig. 4:**
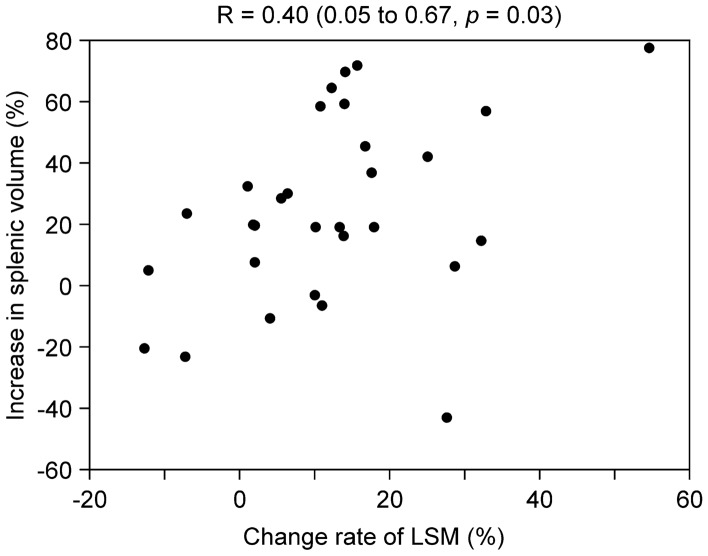
Scatter plots and correlation coefficients for the change in rate of LSM and increase in splenic volume Pearson's correlation coefficients (R 95% Confidence interval, CI) were calculated for the change in rate of liver stiffness measurement (LSM) and increase in splenic volume.

The change in rate of LSM was significantly increased patients with an SV increase ≥ 30% (*p* = 0.04, Table 5). The change in rate of LSM before and after oxaliplatin administration with SV increase were moderately correlated (correlation coefficient: *r *= 0.40; Fig. 4). When the reproducibility of 21 LSMs was examined, the ICC was 0.65. A Bland–Altman analysis indicated concordance between the two examiners (mean difference, 0.03; 95% limits of agreement, − 0.22–0.27).

## Correlation between laboratory data and SV increase

Platelet counts decreased significantly in patients with an SV increase ≥ 30% than in those without (*p* = 0.01, Table 5). APRI was significantly higher in patients with SV increase ≥ 30% than those without (*p* = 0.046, Table 5). Meanwhile, the total bilirubin, AST, ALT, ALP, γ-GT levels were not significantly different between patients with and without an SV increase ≥ 30%.

## Discussion

This study is the first to prospectively examine the relationship between changes in ultrasonography (HokUS-10) and LSM, and oxaliplatin-induced increase in SV on CT. Our findings did not demonstrate a correlation between the HokUS-10 score and SV increase. Oxaliplatin-induced SOS takes longer to develop than classical SOS, which develops within 21 days of HSCT [34]. The cutoff value of HokUS-10 score, useful in diagnosing classical SOS, might have failed to capture small signs of oxaliplatin-induced SOS occurring gradually overtime. Therefore, we did not use the original cutoff for HokUS-10 score and assess the change in rate of continuous parameters to capture the small signs of hepatic congestion. We found weak positive correlation of the change in rates of the right lobe vertical diameter, PV diameter, and moderate positive correlation of the hepatic artery RI with SV increase. When SOS develops, right lobe vertical diameter increases, reflecting hepatomegaly. Furthermore, PV diameter enlargement, hepatic artery RI increase, reflect hepatic congestion. Since these correlations agreed with SOS disease state, they seemed to be appropriate results for identifying early signs of oxaliplatin-induced SOS. For detecting oxaliplatin-induced SOS via ultrasonography, focusing on these three parameters may be better than using any of the HokUS-10 parameters; however, further investigations are required.

Our study revealed that the change in rate of LSM evaluated by SWE and SV increase were moderately correlated. LSM elevation follows liver congestion in patients with heart failure and liver transplantation [40–43]. In this study, elevated LSM may have been affected by liver congestion from oxaliplatin-induced SOS, whereas oxaliplatin-induced SOS may have caused liver fibrosis and increased LSM. Two previous studies assessed LSM in diagnosing oxaliplatin-induced SOS; a laboratory study and another conducted in humans using transient elastography [44, 45]. No studies have investigated the usefulness of SWE to diagnose oxaliplatin-induced SOS in humans, and our study is the first report. Based on the results, LSM evaluated by SWE may be useful for diagnosing oxaliplatin-induced SOS early.

The results of this study allowed us to confirm the inter-examiner reproducibility of LSM evaluated by SWE. Although the inter-examiner reproducibility of HokUS-10 has already been shown [46], no previous reports investigated the reproducibility of LSM by SWE in patients with oxaliplatin-induced SOS.

As for the correlation between laboratory values and SV increase, platelet counts were significantly decreased in patients with SV increase ≥  30% than in those without. Although already reported, platelet count significantly decreases when SOS develops [13, 23, 47, 48]. Our results suggest that thrombocytopenia might occur earlier than SV increase ≥ 30%. The previous studies showed APRI as a predictive indicator for oxaliplatin-induced SOS [49]. This study showed APRI was significantly increased before SV increase ≥ 30% and APRI may be useful for prediction oxaliplatin-induced SOS early as previously reported.

Because oxaliplatin monotherapy is not administered in clinical practice, it is difficult to exclude the influence of anticancer drugs other than oxaliplatin. Although several studies reported that Bevacizumab (BV) alleviates SOS [23], BV impact was difficult to evaluate, because no patient used BV in this study. However, no drugs other than oxaliplatin, including irinotecan and 5-FU has shown association with SV increase. In this study, comparing SV increase with LSM and HokUS-10 parameters, we considered oxaliplatin-induced SOS to be the most suspicious cause of changes in LSM and some parameters.

This study has several limitations, such as its limited sample size, single-center setting, need for training in diagnostic imaging. The SV increase ≥ 30% used as a diagnostic indicator of oxaliplatin-induced SOS in this study does not have high diagnostic ability with sensitivity and specificity of 64% and 71% [23], thus it is necessary to compare ultrasonography and elastography with histopathological diagnosis are also needed in the future. To establish modified scoring system using either or both ultrasonographic parameters and a more accurate cutoff value of LSM, large-scale validation studies are required.

In conclusion, the usefulness of HokUS-10 itself could not be shown; however, some parameters included in HokUS-10 and LSM evaluated by SWE could be useful for the early diagnosis of oxaliplatin-induced SOS. These parameters and LSM could be a new early diagnostic indicator of oxaliplatin-induced SOS.

## Supplementary Information

Below is the link to the electronic supplementary material.

Supplementary file1 (PDF 314 KB)

Supplementary file2 (PDF 190 KB)
