# Supplementary material for: Usefulness of ultrasonography and elastography in diagnosing oxaliplatin-induced sinusoidal obstruction syndrome
Source: Int J Clin Oncol. 2022 Aug 30;27(11):1780–90. doi: 10.1007/s10147-022-02235-4 (PMC9606101; doi:10.1007/s10147-022-02235-4)
Supplement: Supplementary file 1 — Supplementary file1 (PDF 314 KB) [file 10147_2022_2235_MOESM1_ESM.pdf]

Title: Usefulness of Ultrasonography and Elastography in Diagnosing Oxaliplatin-induced Sinusoidal Obstruction Syndrome

Journal: International Journal of Clinical Oncology

Author names: Rika Saito, Yasuyuki Kawamoto, Mutsumi Nishida, Takahito Iwai, Yasuka Kikuchi, Isao Yokota, Ryo Takagi, Takahiro Yamamura, Ken Ito, Kazuaki Harada, Satoshi Yuki, Yoshito Komatsu and Naoya Sakamoto

Corresponding author: Yoshito Komatsu

Division of Cancer Center, Hokkaido University

Hospital Kita-15, Nishi-7, Kita-ku, Sapporo, Japan

E-mail: ykomatsu@ac.cyberhome.ne.jp

Fig. S1 Supplementary data on the images of HokUS-10

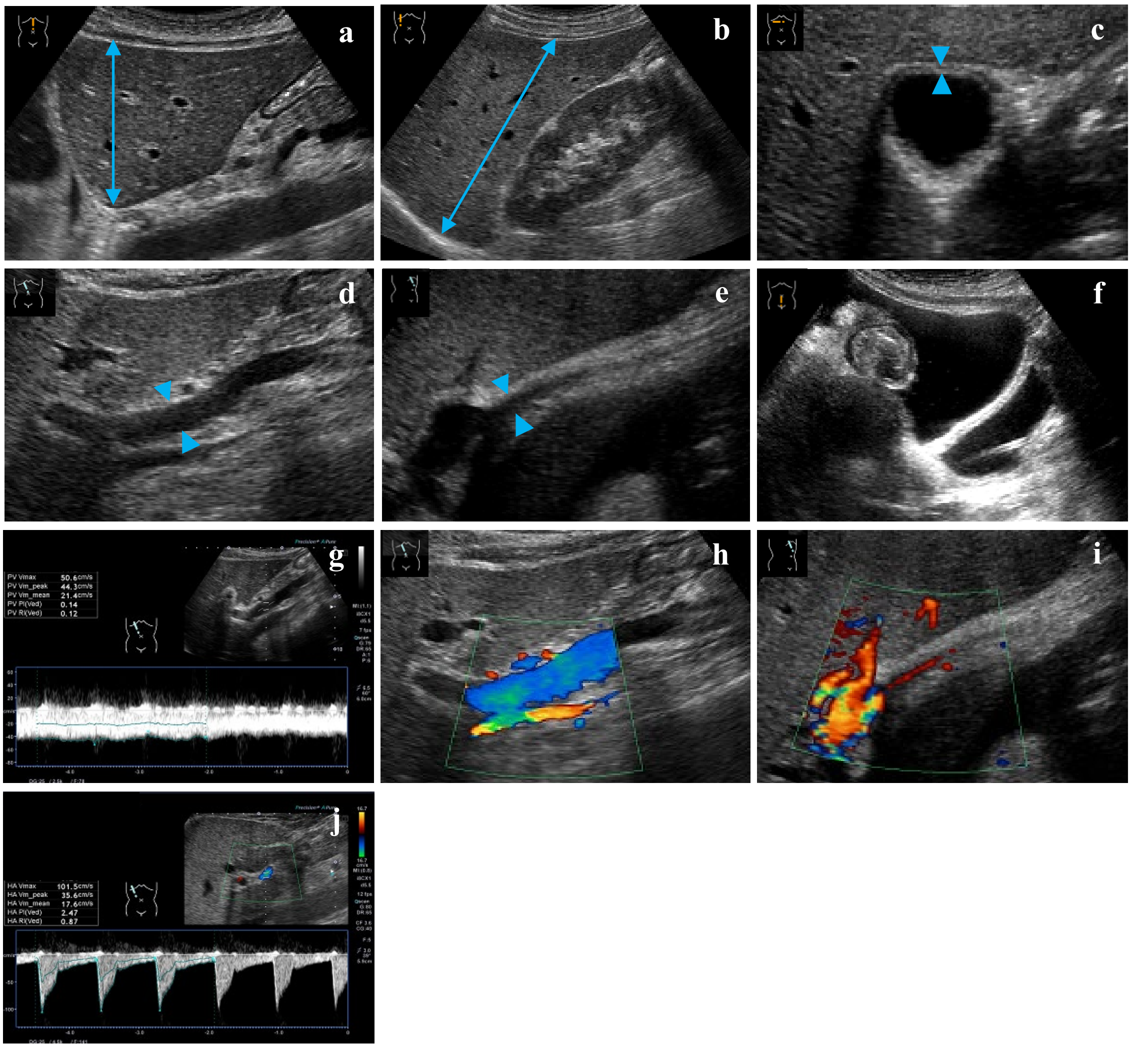

The Hokkaido ultrasonography scoring system (HokUS-10) consists of the following 10 parameters: hepatomegaly in the (a) left lobe vertical diameter and (b) right lobe vertical diameter, (c) gallbladder wall thickening, (d) portal vein (PV) diameter, (e) paraumbilical vein (PUV) diameter, (f) amount of ascites, (g) PV mean velocity, (h) direction of PV blood flow signal, (i) appearance of PUV blood flow signal, and (j) hepatic artery resistive index.
