# Supplementary material for: Usefulness of ultrasonography and elastography in diagnosing oxaliplatin-induced sinusoidal obstruction syndrome
Source: Int J Clin Oncol. 2022 Aug 30;27(11):1780–90. doi: 10.1007/s10147-022-02235-4 (PMC9606101; doi:10.1007/s10147-022-02235-4)
Supplement: Supplementary file 2 — Supplementary file2 (PDF 190 KB) [file 10147_2022_2235_MOESM2_ESM.pdf]

Title: Usefulness of Ultrasonography and Elastography in Diagnosing Oxaliplatin-induced Sinusoidal Obstruction Syndrome

Journal: International Journal of Clinical Oncology

Author names: Rika Saito, Yasuyuki Kawamoto, Mutsumi Nishida, Takahito Iwai, Yasuka Kikuchi, Isao Yokota, Ryo Takagi, Takahiro Yamamura, Ken Ito, Kazuaki Harada, Satoshi Yuki, Yoshito Komatsu and Naoya Sakamoto

Corresponding author: Yoshito Komatsu

Division of Cancer Center, Hokkaido University

Hospital Kita-15, Nishi-7, Kita-ku, Sapporo, Japan

E-mail: ykomatsu@ac.cyberhome.ne.jp

Table S1. Supplementary data on cross-tabulation of each HokUS-10 parameter score

**Left lobe vertical diameter**	≥ 30% increase in splenic volume		Total	**Risk ratio**
	Observed	Not observed	(n)	**(95% CI)**
Score ≥ 1	1	1	2	
Score < 1	11	17	28	**1.27 (0.30-5.48)**
Total (n)	12	18	30	

**Right lobe vertical diameter**	≥ 30% increase in splenic volume		Total	**Risk ratio**
	Observed	Not observed	(n)	**(95% CI)**
Score ≥ 1	3	7	10	
Score < 1	9	11	20	**0.67 (0.23-1.93)**
Total (n)	12	18	30	

**Gallbladder wall thickening**	≥ 30% increase in splenic volume		Total	**Risk ratio**
	Observed	Not observed	(n)	**(95% CI)**
Score ≥ 1	0	0	0	
Score < 1	11	15	26	**-**
Total (n)	11	15	26	

**Portal vein diameter**	≥ 30% increase in splenic volume		Total	**Risk ratio**
	Observed	Not observed	(n)	**(95% CI)**
Score ≥ 1	1	3	4	
Score < 1	11	15	26	**0.59 (0.10-3.42)**
Total (n)	12	18	30	

**Paraumbilical vein diameter**	≥ 30% increase in splenic volume		Total	**Risk ratio**
	Observed	Not observed	(n)	**(95% CI)**
Score ≥ 2	0	0	0	
Score < 2	12	18	30	-
Total (n)	12	18	30	

**Ascites**	≥ 30% increase in splenic volume		Total	**Risk ratio**
	Observed	Not observed	(n)	**(95% CI)**
Score ≥ 1	7	7	14	
Score < 1	5	11	16	**1.60 (0.65-3.92)**
Total (n)	12	18	30	

**Portal vein mean velocity**	≥ 30% increase in splenic volume		Total	**Risk ratio**
	Observed	Not observed	(n)	**(95% CI)**
Score ≥ 1	1	3	4	
Score < 1	11	15	26	**0.59 (0.10-3.42)**
Total (n)	12	18	30	

**Direction of the portal vein blood flow signal**	≥ 30% increase in splenic volume		Total	**Risk ratio**
	Observed	Not observed	(n)	**(95% CI)**
Score ≥ 1	0	0	0	
Score < 1	12	18	30	-
Total (n)	12	18	30	

**Appearance of paraumbilical vein blood flow signal**	≥ 30% increase in splenic volume		Total	**Risk ratio**
	Observed	Not observed	(n)	**(95% CI)**
Score ≥ 2	0	0	0	
Score < 2	12	18	30	**-**
Total (n)	12	18	30	

**Hepatic artery resistive index**	≥ 30% increase in splenic volume		Total	**Risk ratio**
	Observed	Not observed	(n)	**(95% CI)**
Score ≥ 1	8	9	17	
Score < 1	4	9	13	**1.53 (0.59-3.99)**
Total (n)	12	18	30	

We summarized an increase ≥30% in splenic volume and each score of HokUS-10 parameters (left and right lobe vertical diameter, gallbladder wall thickening, portal vein diameter, paraumbilical vein diameter, ascites, portal vein mean velocity, direction of the portal vein blood flow signal, appearance of paraumbilical vein blood flow signal, hepatic artery resistive index) in the form of cross tabulations and calculated the risk ratio (95% Confidence interval, CI).
